# Comparative Studies of c- and m-Plane AlN Seeds Grown by Physical Vapor Transport

**DOI:** 10.3390/ma15248791

**Published:** 2022-12-09

**Authors:** Xiaogang Yao, Zhen Kong, Shengfu Liu, Yong Wang, Yongliang Shao, Yongzhong Wu, Xiaopeng Hao

**Affiliations:** 1 State Key Lab of Crystal Materials, Shandong University, Jinan 250100, China; 2 Department of Materials Science and Engineering, Qilu University of Technology, Jinan 250353, China

**Keywords:** AlN, PVT, structure parameter, compressive stress

## Abstract

The ultra-wide bandgap semiconductor AlN has attracted a great deal of attention owing to its wide application potential in the field of electronics and optoelectronic devices. In this report, based on the mechanism of the physical vapor transport (PVT) growth of AlN crystal, the c- and m-plane AlN seed crystals were prepared simultaneously through special temperature field design. It is proved that AlN crystals with different orientations can be obtained at the same temperature field. The structure parameter of AlN crystal was obtained through the characteristic evaluations. In detail, XPS was used to analyze the chemical states and bonding states of the surface of seed crystals. The content of oxygen varied along with distinct orientations. Raman spectrum documented a small level of compressive stress on these crystal seeds. Tested results confirmed that the prepared AlN crystal seeds had high quality.

## 1. Introduction

Semiconductor materials have important applications in extreme environments, solid-state lighting and displays, short-wavelength lasers, and optical detection [1,2,3,4]. As an ultrawide bandgap semiconductor material, AlN has an ultra-wideband gap of up to 6.2 eV which gives it the ability to remain transparent in the deep UV region (<200 nm). It is widely used in many fields, such as ultraviolet disinfection, water purification, gas sensing and ultraviolet curing [5,6,7]. In addition, because of its unique physical and chemical properties, it has an important presence in the fields of surface acoustic waves, high-frequency devices, high-voltage devices or high-power devices, and so on [8,9,10,11,12]. More importantly, it is also a favorable substrate for alloys AlGaN, InGaN, and AlGaInN. Compared with the commonly used sapphire substrate, AlN substrates have much smaller lattice mismatch with AlGaN, InGaN, and AlGaInN, which can effectively reduce dislocation density [8,13,14,15]. Furthermore, the demonstration of these properties needs to be based on the convenient use of AlN single crystal substrates with reliable size and quality.

Although AlN crystals can be grown by hydride vapor phase epitaxy (HVPE) [13,16], metal organic chemical vapor deposition (MOCVD) [6], molecular beam epitaxy (MBE) [17], physical vapor transport (PVT) [3,5,8,18,19,20] or other methods, it has been proved that the PVT method is the best method to obtain bulk AlN crystals [21]. Two approaches are proposed to grow AlN crystals by the PVT method. One approach is to grow AlN crystals using the 4H/6H-SiC substrate due to their similar thermal expansion coefficients and lattice constants [5,18,22]. This approach can quickly produce large-size AlN crystals. However, since the sublimation of heterogeneous substrates at high temperature, the AlN crystals contain high content of C, Si and other unintentional impurities, which seriously affect the optical and the structural quality of crystals. Another approach is spontaneous nucleation on W substrates, followed by repeated growth to obtain larger-size AlN crystals [8,20,23]. The AlN crystals obtained by this method have high crystal quality due to the low concentration of unintentional doped elements. 

Existing studies show that the nucleation of AlN crystal can be controlled effectively by controlling the supersaturation and temperature distribution in the nucleation region, so as to obtain independent seed crystals [24,25,26,27]. However, because the PVT method is usually performed over 2100 °C and the crystal material is anisotropic, the control of nucleation is very difficult. Hartmann et al. successfully prepared independent AlN seeds by high-temperature nucleating with a tungsten porous plate in the middle of the TaC crucible to reduce supersaturation in the middle of the crucible [26]. Chen et al. successfully reduced the nucleation density by adjusting the direction of temperature gradients during the growth process and obtained independent AlN seeds on W substrate [27]. Since the composition and stress can seriously affect the optical and structural quality of AlN crystal, it has great practical significance and scientific value to understand the composition and stress level of the initial AlN seeds.

Herein, independent AlN seeds were prepared by controlling temperature gradient and supersaturation value during nucleation. These grains were controlled by a special growth process to a small size, which better reflects the initial stage of spontaneous nucleation of AlN seeds on W substrate. In order to understand the initial crystal structure and stress level, the comparative characterization of the obtained individual seeds was also carried out. This work will deepen our understanding of the AlN crystals.

## 2. Experimental Section

Self-nucleated AlN seeds were grown using physical vapor transport (PVT) in a self-designed induction heating system in which the temperature field and AlN source were provided. Carbon composite material was used as insulation material, the pressure was controlled between 800 and 1200 mbar during the growth process, the temperature was controlled at 2150 °C, and the growth environment was filled with high-purity nitrogen (99.99%). The temperature at the center position of the crucible cover was determined by the infrared thermometer (RAYMR1SCSF, Raytek, Santa Cruz, CA, USA). The AlN source was obtained from commercially AlN powder after being heated at 20 h at 1800 °C and 15 h at 2100 °C. The crucible apparatus was made of tungsten. The nucleation region was arranged inside the crucible, and the distance from the bottom was 2/3 of the height of the crucible. All tungsten products were cleaned with acetone, ethanol, and deionized water before heating.

Single-crystal XRD data were collected through a Bruker SMART APEX II diffractometer equipped with a 4 K CCD area detector using graphite monochromatic Mo Kα radiation (λ = 0.71073 Å). The structure was solved by Patterson synthesis using SHELXS97 and refined by the full-matrix least-squares program SHELXL97. X-ray photoelectron spectroscopy (XPS) was obtained by Thermo ESCALAB 250. Raman spectra were determined with a 532 nm laser by using a LabRAM HR800 Raman spectrometer (Horiba Jobin Yvon, Edison, NJ, USA).

## 3. Results and Discussion

**Figure 1:**
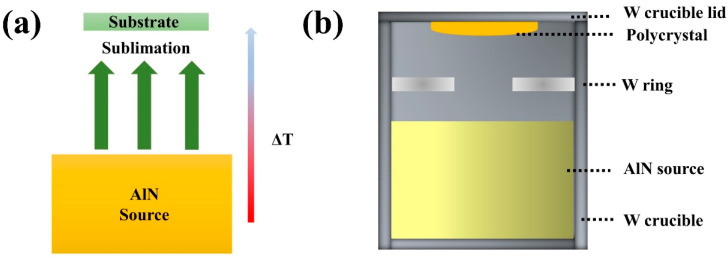
The illustration of the PVT method (**a**) and schematic diagram of the preparation process of AlN seeds (**b**).

Figure 1a shows the diagram of the preparation process of AlN crystals by use of the PVT method. The preparation process of AlN crystal by the PVT method can be simply summarized as sublimation and recondensation. Commercial AlN powders were purified twice at high temperatures and utilized as sources in the general preparation of AlN crystals. First, in a nitrogen atmosphere, AlN source was sublimated at high temperature to produce vapor Al and N_2_. Afterword, these vapors moved from high to low temperature in the direction of a temperature gradient (ΔT). Finally, those vapors continuously collided, diffused, and were absorbed and desorbed on the substrate. The whole process can be briefly described by the following reaction equation [5,19]:

$$\mathrm{AlN}(s)\;\to \;\mathrm{Al}(v)+\frac{1}{2}N_{2}(v)$$

$$\mathrm{Al}(v)+\frac{1}{2}N_{2}(v)\;\to \;\mathrm{AlN}(s)$$

Obviously, vapor Al and N_2_ are the key species for the growth of AlN crystal by the PVT method. Since nitrogen acts as the environmental atmosphere in the growth process, it is obvious that vapor Al is the most critical species to control the growth [28]. Supersaturation directly affects the nucleation density and growth rate of crystal on the growth surface [26,27,29]. However, reasonable supersaturation is a necessary condition to control the nucleation of AlN grains, because supersaturation that is too low will reduce the nucleation probability and lead to a slow growth rate, while supersaturation that is too high will lead to high nucleation probability and a fast growth rate. Existing studies have shown that, for nucleation of AlN crystals, the supersaturation should be controlled between 2.5 and 3 in order to obtain small nucleation density with independent nucleation and non-interference of the crystal’s nucleus, and ensure the growth rate is within the effective range (>200 μm/h) [27,30].

A diagram of a crucible for growth is shown in Figure 1b. The crucible is cylindrical with a bottom, and an AlN source was placed inside the crucible to provide material transfer during growth. A crucible cover was arranged above the crucible, and its thickness was consistent with the thickness of crucible’s bottom. The crucible cover and crucible together provided a chamber for crystal growth. During the growth process, the center of the crucible cover was polycrystalline because of the lower temperature. The nucleation region was a tungsten ring.

**Figure 2:**
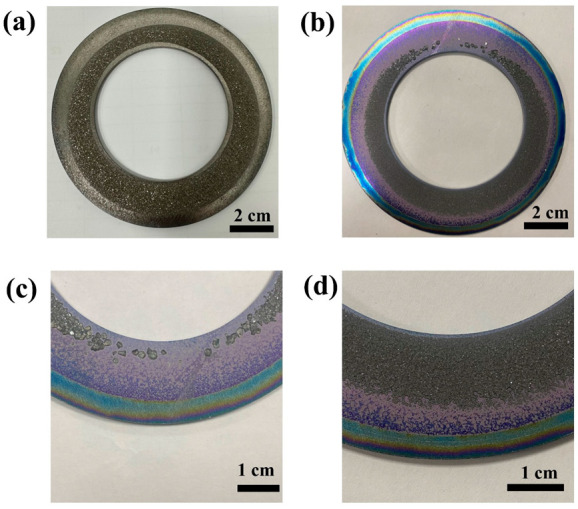
The photographs of the tungsten ring after growth without control (**a**) and with control (**b**), enlargement of the top (**c**) and bottom (**d**) of Figure b.

The distribution of the supersaturation and temperature gradient in the nucleation region was regulated by changing growth techniques. In Figure 2a, the crystals growing on the ring pushed against each other. In Figure 2b, independent seeds were obtained on one side of the ring, while the polycrystalline nucleation region was still on the other side. In particular, it can be seen from Figure 2b that the distribution area of the crystal on the ring gradually decreases from one side to the other, and the distribution is symmetric. Finally, independent seeds were obtained on the upper side of the ring. Figure 2c shows the enlarged view of the independent nucleation region. It can be seen that the nucleation density of crystals is extremely low. In addition, the independent seeds had a relatively small and consistent size, about 1~2 mm. Those grains were independent of each other and retained the shape of the primary crystal, and the original growth plane of the crystal was preserved. All the seed crystals were about 500 μm thick, and independent of the growth plane. This indicates that the crystal growth rate is mainly controlled by the thermal designed in the growth crucible, rather than by crystallographic differences [20]. Correspondingly, in Figure 2d, the nucleation density is very high, and grains are squeezed against each other. Most grains lost their original crystal shape. This special phenomenon benefits from the combination between the crucible structure and the ring. By changing the tilt angle of the ring, the temperature field and supersaturation distributed uniformly on the ring were changed. It effectively changes the distribution of supersaturation and the temperature gradient in different regions of the ring. As a result, the supersaturation and temperature gradient on the upper side of the ring (upper side of Figure 2b) decrease, and the supersaturation and temperature gradient on the lower side of the ring (lower side of Figure 2b) increased. In particular, unnecessary nucleation at lower temperatures was inhibited by controlling the pressure in the growth environment prior to the nucleation temperature. The synergistic effect of the special crucible design and unique growth process resulted in an independent smaller grain size [8,23,26,27].

**Figure 3:**
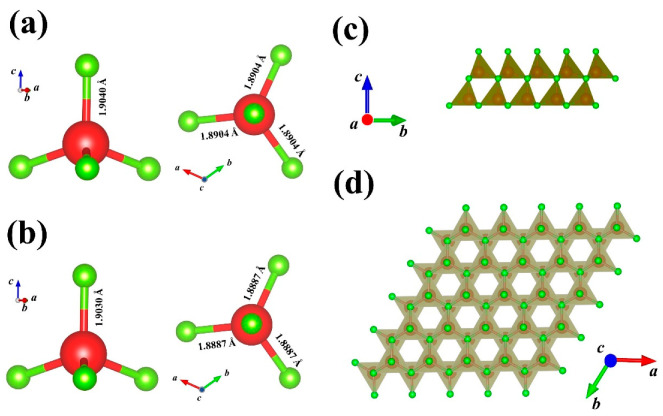
The molecular structure of the seeds of c-plane (**a**) and m-plane (**b**), and the crystal structure at different viewing angles (**c**,**d**).

The crystal structure was analyzed to understand the growth structure of different seeds. The crystal structure of the samples was determined by X-ray single crystal diffraction. AlN crystallizes in the hexagonal polar space group of *P*_63_, of which structure consists of Al-N_4_ tetrahedra connected by Al-N. In theory, the four Al-N bonds of AlN crystal are of the same length, but the actual Al-N bond lengths are slightly different due to different growth patterns and internal stresses. As shown in Figure 3a, b, the length of three Al-N bonds in the AlN seed growing in the c-plane is 1.8904 Å, the other is 1.9040 Å. The length of three Al-N bonds in the AlN seed growing in the m-plane is 1.8887 Å, the other is 1.9030 Å. Figure 3c, d shows the crystal structure at different viewing angles.

**Figure 4:**
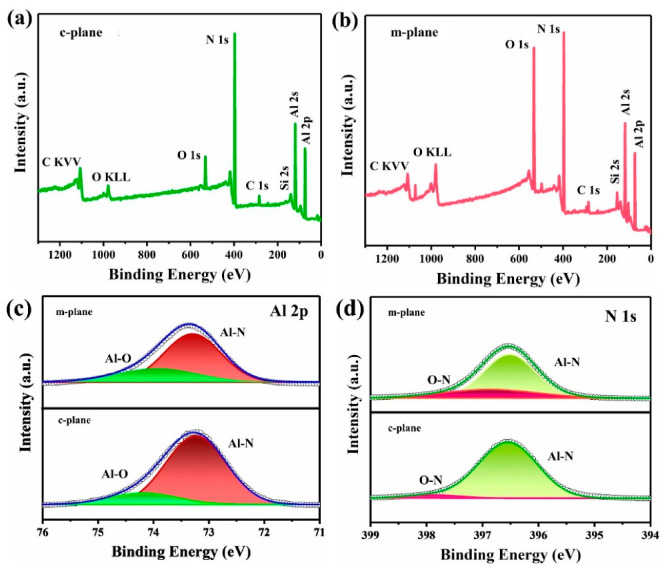
XPS survey of the c-plane (**a**) and m-plane (**b**), and Al 2p (**c**) and N 1s (**d**) high-resolution XPS spectra of the c-plane and m-plane.

In order to further investigate the element types and valence states, the prepared AlN seed samples were subjected to XPS analysis. The investigation scan from XPS is shown in Figure 4a,b, where it can be clearly seen that these AlN seeds are composed of aluminum, nitrogen, carbon, oxygen and silicon elements. Moreover, the relative area of the O 1s peak in m-plane is larger, indicating that the content of oxygen impurities is higher. Previous studies have shown that oxygen impurity plays an important role in the growth and transformation of AlN crystals [7]. The occurence of the carbon element peak because of carbonaceous impurities in the growth system was adsorbed to the surface of the sample. High-resolution XPS spectra of Al 2P and N1S peaks of samples are shown in Figure 4c,d. By screening the peaks of N 1s and Al 2p, two chemical states of aluminum and nitrogen with different binding energies (B.E.) can be obtained. From the Al 2p core-level XPS (Figure 4c) sample, it can be seen that there are two characteristic peaks, which are Al-N (73.23 eV), Al-O (74.18 eV) for the c-plane and Al-N (73.29 eV), Al-O (73.92 eV) for the m-plane. From the XPS high-resolution spectrum of N 1s, there are two distinct characteristic peaks, which are the Al-N (396.54 eV), O-N (397.89 eV) for the c-plane, and Al-N (396.51 eV), O-N (396.88 eV) for the m-plane. Al 2P peaks of all samples are dominated by Al-N, while N 1s peaks are dominated by N-Al [6]. In general, if one element loses electrons, its binding energy will be transferred to a higher field, and if an element gains an electron, its binding energy will transfer to a lower field. For the XPS spectra of Al 2p and N 1s, the chemical B.E. of the c-plane is greater than the m-plane. This may be due to the higher content of oxygen in the m-plane causing the element to lose more electrons, resulting in charge transfer, which makes the binding energy of the element transfer to the lower field. The surface oxide thickness, *T* (nm), can be estimated from the Al 2p oxide to the metal peak ratio (*I_0_/I_m_*) using the equation [31,32].

$$T\left(nm\right)=\lambda_{0}\sin\theta \ln\left(\frac{N_{m}I_{0}\lambda_{m}}{N_{0}I_{m}\lambda_{0}}+1\right)$$

where *N_m_/N_0_* = 1.6 is the ratio of the volume densities of aluminum atoms in metal to oxide, *λ_m_* and *λ_0_* are the inelastic mean free path of alumina and nitrogen, *θ* = 90°. The *T*_seed1_ = 6.79 nm, while the *T*_seed2_ = 13.78 nm.

**Figure 5:**
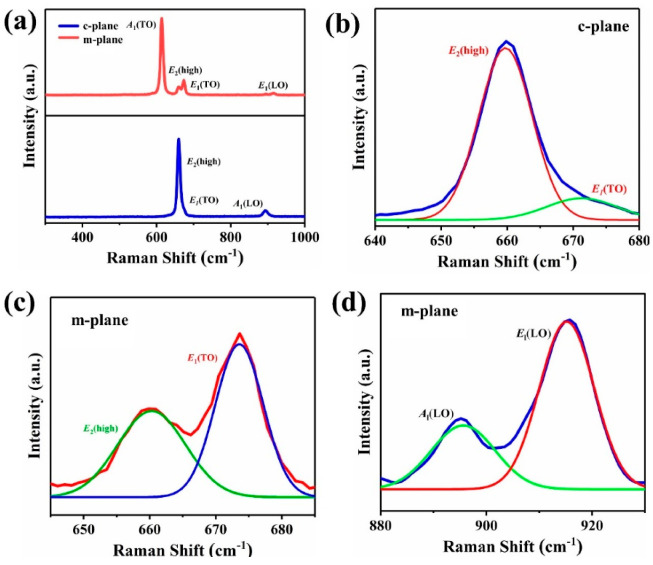
Raman spectrum of the samples (**a**) and the fitting Raman peaks of the c-plane (**b**) and m-plane (**c**,**d**).

Phonon properties determine the thermal and optical properties of crystals. It is important to understand the lattice dynamics of AlN seeds for the application of AlN substrates. Raman spectrum allows direct, lossless and effective measurement of the phonon spectral characteristics of semiconductors. The hexagonal wurtzite structure of AlN belongs to the C_6v_ space group. Six active Raman modes are predicted by group theory: *E*_2_(low) (at 248.6 cm^−1^), *A*_1_(TO) (at 611.0 cm^−1^), *E*_2_(high) (at 657.4 cm^−1^), *E*_1_(TO) (at 670.8 cm^−1^), *A*_1_(LO) (at 890.0 cm^−1^) and *E*_1_(LO) (at 912.0 cm^−1^) modes [33]. However, the growth surfaces of the crystals are different, and the anisotropy of the crystals results in different activity modes in the Raman spectrum. The prepared seed crystals are characterized by Raman spectrum at room temperature, as shown in Figure 5a, showing the Raman spectra of seeds. The observed phonon modes in the Raman spectra of AlN seeds obey the C_6v_ point group symmetry rules for the corresponding scattering configuration. The *E*_2_(high) (at 659.74 cm^−1^), *E*_1_(TO) (at 671.28 cm^−1^) and *A*_1_(LO) (at 893.13 cm^−1^) modes are observed in the c-plane, corresponding, the *A*_1_(TO) (at 614.13 cm^−1^), *E*_2_(high) (at 660.29 cm^−1^), *E*_1_(TO) (at 673.56 cm^−1^), *A*_1_(LO) (at 895.64 cm^−1^) and *E*_1_(LO) (at 915.22 cm^−1^) modes are observed in the m-plane. The AlN phonon anisotropy is high, with the *A*_1_(TO) mode was weaker in highly oriented c-orientation crystal and stronger in highly oriented m-orientation crystal, and the *E*_2_(high) mode being stronger in high c-orientation crystal and weaker in highly oriented m-orientation crystal. Compared with the c-plane, m-plane shows richer Raman modes information. In the c-plane, only *E*_2_(high) and *A*_1_(LO) are displayed, while *E*_2_(low), *A*_1_(TO), *E*_1_(TO) and *E*_1_(LO) are prohibited. In the m-plane, abundant Raman modes are displayed, and only *E*_2_(low) is disabled. Remarkably, neither seed crystal had the Raman peaks not predicted by group theory. The high consistency of the peak position proves that the prepared seeds have good crystal quality and low impurity content. This advantage is due to the higher growth temperature and purer growth environment of spontaneous nucleation seeds.

The Raman spectrum can not only characterize phonon spectral characteristics, but also reflect the quality and structural characteristics of crystal [34,35,36]. In order to better understand the quality and structural characteristics of the prepared seeds, the observed Raman modes are compared with other work (Table 1). As mentioned above, AlN crystal has strong phonon anisotropy, so the number of Raman modes varies with the growth surface. The Raman phonon energies (frequencies) of all the observed modes are comparable to the results reported for nearly unstrained AlN. Meanwhile, all the Raman modes reveal a slight blueshift compared to the unstressed AlN, which reflects the slight compressive stress on the growth surface.

**Figure 6:**
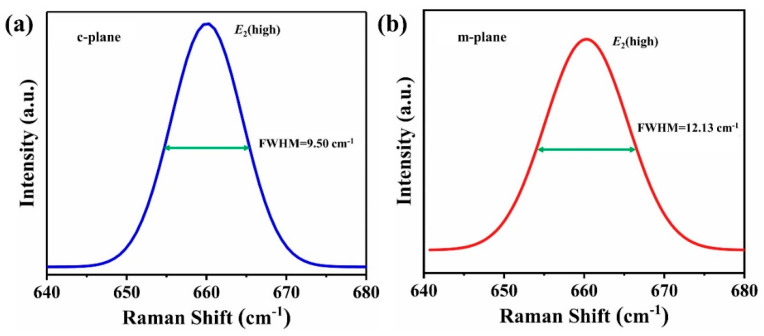
The FWHM of *E*_2_(high) mode of the c-plane (**a**) and m-plane (**b**).

In order to further understand the quality of the seeds, Gaussian fitting was performed on the Raman spectrum, as shown in Figure 6. As can be seen from the Figure 6, the full width at half maximum (FWHM) of *E*_2_(high) modes is narrow, and the FWHM of c-plane and m-plane is 9.50 cm^−1^ and 12.14 cm^−1^, respectively. The observed FWHM of *E*_2_(high) modes is compared with other work [5,27,37], the results are presented in Table 2. It is generally assumed that the FWHM of the Raman peak reacts to the crystallinity of the material and material defects [36]. It is confirmed that the seeds have high structure quality. The *E*_2_(high) Raman mode is highly sensitive to stress and is often used to judge the stress level in AlN crystals. In particular, the following formula can be used to calculate the stress and strain levels of the crystal [5,20,27]:

$$\delta =\frac{\Delta \omega }{k}$$

where the *k* is the *E*_2_(high) Raman stress factor, ∆ω is the shift of the frequency, and the *δ* is the stress.

$$\varepsilon m_{\;}=\frac{\Delta \omega }{\left(2\alpha +\beta \right)}$$

where the *α* and *β* are deformation potentials of the *E*_2_(high) phonon, *ε_m_* is the mean strain.

For the hexagonal AlN crystal, *k* = −6.3 ± 1.4 cm^−1^ GPa^−1^, *α* = −1044 ± 31 cm^−1^ and *β* = −1140 ± 96 cm^−1^ [5,20,27,38]. If assume that the shift in the peak position is due to strain, a compressive stress of 0.39 ± 0.09 GPa and a strain of 7.25 ± 0.33 × 10^−4^ in c-plane, and a compressive stress of 0.49 ± 0.09 GPa and a strain of 8.95 ± 0.33 × 10^−4^ in m-plane can be obtained.

## 4. Conclusions

In summary, c-plane and m-plane AlN seeds were prepared through adjusting the growth techniques by use of the PVT method. Furthermore, the crystal structure was determined by X-ray single crystal diffraction. In particular, the imparity oxygen content in crystals with different orientations was observed by XPS analysis. Moreover, the stress level in the crystals was analyzed by Raman spectrum. This work will assist us to deepen our comprehension of AlN crystals’ growth.

## Figures and Tables

**Table 1:** Raman phonon energies (cm^−1^) of the prepared AlN seeds.

Phonon Symmetry	c-Plane	m-Plane	PVT on SiC [5]	PVT on W-Substrate [27]	CHVPE on a-Al_2_O_3_ [27]	Free-Stress [33]
*E*_2_(low)	-	-	247.4	239	249	248.6
*A*_1_(TO)	-	614.13	---	610	611	611.0
*E*_2_(high)	659.74	660.29	655.3	658	657	657.4
*E*_1_(TO)	671.28	673.56	-	670	671	670.8
*A*_1_(LO)	893.13	895.64	902.7	-	890	890.0
*E*_1_(LO)	-	915.22	-	914	912	912.0

**Table 2:** The FWHM of *E*_2_(high) modes (cm^−1^) of the prepared AlN seeds.

	c-Plane	m-Plane	[5]	[27]	[37]
FWHM	9.50	12.13	~40	11, 12	18–22, 23

## Data Availability

The data presented in this study are available upon request from the corresponding author. The data are not publicly available due to privacy.
